# Burrata and the Bean: A Rare Case of *Lactobacillus casei* Endocarditis Complicated by Rapidly Progressive Glomerulonephritis

**DOI:** 10.1155/crdi/7709602

**Published:** 2026-05-28

**Authors:** Jason Chavez, Joseph Borick, Fernando Pedraza Taborda, Paola Lichtenberger

**Affiliations:** ^1^ Department of Infectious Diseases, Miami Veterans Affairs Medical Center, Miami, Florida, USA, va.gov; ^2^ Department of Nephrology, Miami Veterans Affairs Medical Center, Miami, Florida, USA, va.gov

**Keywords:** endocarditis-associated GN, glomerulonephritis, infective endocarditis, *Lactobacillus casei*, rapidly progressive glomerulonephritis, renal failure

## Abstract

Infective endocarditis occurs when there is an infection of the heart valves or associated cardiac hardware/devices. While typical organisms include *Staphylococcus* and *Streptococcus* species, there are reports of less common organisms such as *Lactobacillus* species as causative agents as well. Such infections can lead to complications, including renal failure from rapidly progressive glomerulonephritis. A 79‐year‐old male presented with acute heart failure exacerbation in the setting of new severe mitral regurgitation. His acute on chronic kidney disease was attributed to his heart failure exacerbation and returned to baseline after diuresis on discharge. He had recurrent exacerbations with worsening renal function requiring hemodialysis and prompting a renal biopsy that showed rapidly progressive crescentic glomerulonephritis. While infectious causes were not initially suspected, blood cultures were drawn to evaluate this as a potential cause, which persistently grew *Lactobacillus casei*. The infectious diseases team was consulted, and the patient was treated with renally adjusted ampicillin, with repeat blood cultures returning negative. Immunosuppressive therapy was deferred due to concern for active infection, and the patient was treated with targeted antimicrobial therapy alone. This case illustrates an atypical and rare presentation of *Lactobacillus* infective endocarditis with rapidly progressive glomerulonephritis, as well as the balance one must make between the use of antibiotics versus immunosuppression in such patients.

## 1. Introduction

Infective endocarditis (IE) is a serious infection involving the endocardium or prosthetic heart valves and is associated with significant morbidity and mortality [1]. While most cases (75%) are caused by Gram‐positive cocci such as staphylococci, streptococci, and enterococci, less common organisms can occasionally be implicated and may present diagnostic challenges [1].

*Lactobacillus*, a Gram‐positive facultative anaerobic bacterium is a common inhabitant of the oral cavity, gastrointestinal, and genitourinary systems. Widely regarded as low virulence, they are often used in probiotic formulations in lyophilized form or as a fermented food product [2–17]. *Lactobacillus* rarely exhibits pathogenicity, rather offering protection against other pathogens and having positive benefits on colonic health [18]. Despite their benign behavior, *Lactobacillus* species have been reported as rare causes of invasive infections, including bacteremia and endocarditis, particularly in patients with underlying comorbidities or structural heart disease. In a review of 200 cases of *Lactobacillus* infections, it was found that cancer, diabetes, and liver transplant were the more common underlying conditions associated with *Lactobacillus* bacteremia or localized infections [3–17]. The most common species associated with *Lactobacillus* endocarditis is *L. casei*, followed by *L. rhamnosus* and *L. plantarum*. Predisposing risk factors included structural heart disease, dental procedures or underlying dental conditions, and heavy dairy consumption. Mortality associated with *Lactobacillus casei* endocarditis has been documented at around 23% [2].

IE is associated with many complications; these include cardiac, metastatic, neurologic, musculoskeletal, and pulmonary complications. Renal complications are well recognized in IE and encompass a spectrum that includes infarction, interstitial nephritis, and glomerulonephritis (GN). Acute renal failure has also been seen in about one‐third of patients with IE [1]. Rapidly progressive GN (RPGN) represents a severe and uncommon manifestation, characterized by rapid decline in renal function and crescent formation on biopsy.

IE was thought to cause GN through embolism or abscess formation. It is now believed that an immunologic mechanism is at play, as the generation of immune complexes has been documented in prior cases of IE [19]. The pathogenesis is thought to involve immune complex deposition and complement activation, although the clinical presentation can overlap with primary vasculitic processes, complicating diagnosis and management [20]. Up to 10% of patients with pauci‐immune GN have seronegative workup on presentation. Furthermore, renal‐associated pathology and survival were poorer in the ANCA‐positive populations [19, 21]. While immunosuppressive therapy is often indicated for primary vasculitis, it may be harmful in the presence of active infection.

Here we present a case of RPGN‐related IE caused by *Lacticaseibacillus casei* in a patient with new mitral regurgitation (MR) with atypical symptoms, treated with antimicrobials without the need for chronic kidney replacement therapy or immunosuppressants.

## 2. Case Presentation

A 79‐year‐old male with a past medical history of atrial fibrillation (Afib), pulmonary embolism (PE), chronic kidney disease (CKD) stage 3B, newly diagnosed heart failure with preserved ejection fraction (HFpEF), and new severe MR was admitted to the hospital for worsening dyspnea and fatigue.

Prior to his illness, he was independent in his activities of daily living, walking up to 1 mile per day. He was initially evaluated at his local Veterans’ Affair (VA) facility for a 2‐week history of worsening dyspnea. Computed tomography (CT) of the chest was negative for PE or other pulmonary etiology. Nuclear stress testing was unrevealing as well. Cardiac imaging, including transthoracic (TTE) and transesophageal echocardiography (TEE), revealed a myxomatous mitral valve with ruptured chordae tendineae and severe MR. His renal function was at his baseline at the time. Cardiology recommended transfer to a tertiary center for further evaluation and consideration of mitral valve repair.

**FIGURE 1:**
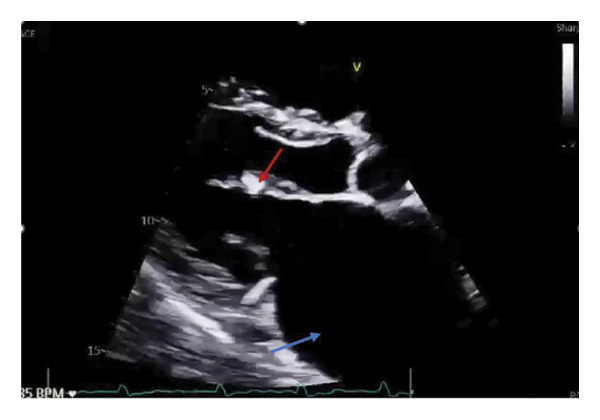
Transthoracic echocardiogram, parasternal long axis view. Note the thickened mitral leaflets (red arrow) and increased size of the left atrium (blue arrow).

**FIGURE 2:**
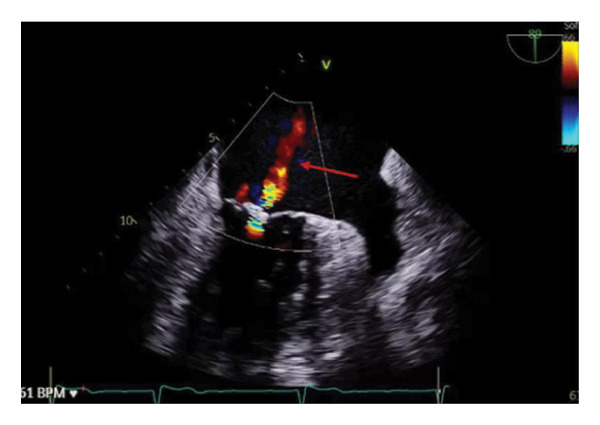
Transesophageal echocardiogram. Mid‐esophageal long axis view. Note the eccentric jet of mitral regurgitation on Doppler directed posteriorly (red arrow). Again seen in the thickened mitral valve and enlarged left atrium.

He was admitted for cardiothoracic evaluation but had progressive worsening of his renal function (increase in creatinine levels from 1.3 mg/dL ‐> 1.4 ‐> 1.6). Urinalysis with urine protein 100 mg/dL, large blood, > 182 red blood cells (RBC) per high‐powered field (HPF), and 5 RBC casts per low‐powered field (LPF). His kidney injury was attributed to cardiorenal syndrome, and he was treated with diuresis. His empagliflozin was held. He had another TTE (Figure 1) and TEE (Figure 2) which demonstrated mild to moderate MR, a severely dilated left atrium (LA), flail A2 scallop of anterior leaflet, and mild prolapse of the P2 scallop of the posterior leaflet. A follow‐up cardiac MRI corroborated moderate to severe MR and a severely dilated LA. Given his worsening renal function and oliguria, mitral repair was deferred by cardiology until this was corrected. Further diuresis was discontinued. He was discharged with outpatient follow‐up for an interval mitral clip to address his new MR.

**FIGURE 3:**
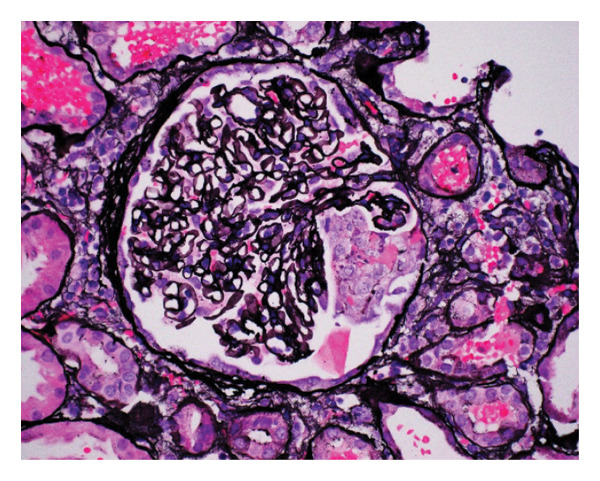
Renal biopsy. Light microscopy, Jones Methenamine Silver stain. Focal necrotizing/crescentic glomerulonephritis with acute tubular injury and numerous RBC casts. Minimal interstitial fibrosis (at most 10%). The glomerulonephritis is characterized by necrosis or acute/cellular crescents in 3 of 7 glomeruli.

**FIGURE 4:**
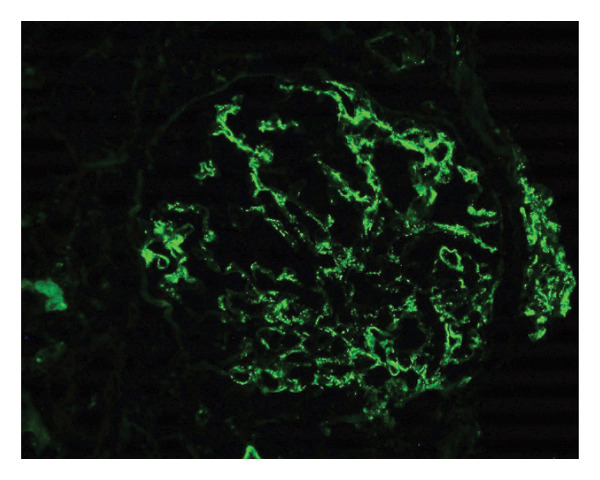
Renal biopsy. Immunofluorescence microscopy. IgM dominant glomerular immune complexes. ANCA negative.

In the interim, he became progressively dyspneic on exertion and further oliguric with darker‐colored urine and decreased oral intake, requiring re‐admission. A detailed timeline of clinical events is provided in Table 1. On admission, he was afebrile; his blood work was notable for a creatinine of 5.3 mg/dL, brain natriuretic peptide (BNP) of 19,000 pg/mL, potassium of 5.6 mEq/L, International Normalized Ratio (INR) of 1.3. No leukocytosis was noted. Chest radiograph noted an enlarged cardiac silhouette. Urinalysis with large blood, urine protein 100 mg/dL, > 182 RBC per HPF, and 5 RBC casts per LPF. No nitrites or leukocyte esterase seen. The microalbumin‐creatinine ratio was 329.4 mg/g. Renal ultrasound showed diffuse increased echogenicity of bilateral renal parenchyma consistent with chronic medical renal disease. Nephrology was consulted for the worsening renal function in the setting of oliguria. Creatinine continued to rise to 7.4 mg/dL on Day 7 of admission, and the patient was started on hemodialysis via a right internal jugular central line. He had a repeat TTE that was largely unchanged from his prior echocardiograms and continued to show severe MR. A kidney biopsy was recommended at this point. Pathology from this revealed focal necrotizing crescentic GN with minimal interstitial fibrosis (Figure 3) and IgM‐dominant immune complexes (Figure 4). Rheumatology was consulted, and a vasculitis workup was initiated (Table 2). Given the setting of new MR, IgM complexes with GN, rheumatology and nephrology recommended an IE workup. The patient was not yet on antibiotics, as his presentation did not initially suggest an infectious etiology.

**TABLE 1:** Clinical timeline of presentation, diagnosis and management.

Date	Clinical events
Week ‐2 to 0	Initial admission for dyspnea; workup negative for pulmonary etiologies; Echocardiograms revealed new severe mitral regurgitation
Initial Admission	Admitted for initial surgical evaluation; mild AKI due to cardiorenal syndrome; discharged with plan for mitral valve repair
Readmission	Readmission with worsening dyspnea, oliguria, poor oral intake
Hospital Day 2	Persistent AKI with hematuria and RBC casts; nephrology consulted and renal biopsy done on Day 5 of admission
Day 7	Renal failure progresses; hemodialysis initiated
Day 8	Renal biopsy results: Focal Necrotizing Crescentic GN with IgM‐dominant immune complexes; Nephrology and Rheumatology recommended IE workup
Day 9	Blood cultures drawn and positive for Gram‐positive rods; Initiated empiric vancomycin and ceftriaxone. Later speciates to *Lactobacillus casei* in 2 of 2 bottles
Day 11	Repeat Blood cultures drawn and grow *Lactobacillus casei* in 2 of 2 bottles; transitioned to ampicillin‐sulbactam
Day 13	Repeat blood cultures negative; immunosuppression deferred
Day 24	Antibiotics narrowed to IV ampicillin based on sensitivities
Day 25	Last hemodialysis session
Day 38	Patient discharged to rehab with clinical improvement. Tentative plans for Mitral valve repair as an outpatient

*Note:* GN = glomerulonephritis.

Abbreviations: AKI = acute kidney injury, IE = infective endocarditis, RBC = red blood cell.

**TABLE 2:** Immunologic and serologic glomerulonephritis workup.

Test	Value	Reference value
ANA	Negative	< 1:40
c‐ANCA	< 1.0 AI (autoantibody)	< 1.0 AI
p‐ANCA	< 1.0 AI	< 1.0 AI
C3	75 mg/dL (low)	90–180 mg/dL
C4	13 mg/dL	10–40 mg/dL
Cryoglobulin	Negative	Negative
Rheumatoid Factor	Positive (1:8) (high)	< 1:8
Anti‐GBM antibody	< 1.0 AI	< 1.0 AI
IgG	1563	700–1600 mg/dL
IgA	169	70–400 mg/dL
IgM	259 mg/dL (high)	40–230 mg/dL
Kappa Free Light Chain	151 mg/L (high)	2.37–20.73 mg/dL
Lambda Free Light Chain	98.7 mg/L	4.23–27.69 mg/dL
Kappa/Lambda Ratio	1.52	0.26–1.65
Hepatitis B core antibody	Negative	Negative
Hepatitis B surface antigen	Negative	Negative
Hepatitis C antibody	Negative	Negative
HIV antibody	Negative	Negative
Rapid Plasma Reagin (RPR)	Nonreactive	Non‐reactive
QuantiFERON (IFN‐y release assay)	Negative	Negative
Strongyloides IgG	Negative	Negative
Bartonella antibody	Negative	Negative
Parathyroid Hormone (PTH)	46 pg/mL	15–65 pg/mL
Aldosterone	4 ng/dL	3–6 ng/dL
Renin	0.55 ng/mL/h	0.25–5.82 ng/mL/h
Iron	32 μg/dL	61–157 μg/dL
Ferritin	206 ng/mL	30–400 ng/mL
Total Iron Binding Capacity (TIBC)	195 μg/dL	149–503 μg/dL
Lactate Dehydrogenase (LDH)	281 U/L (High)	135–225 U/L
Haptoglobin	93 mg/dL	30–200 mg/dL
Thyroid Stimulating Hormone (TSH)	0.90 μIU/mL	0.27–4.20 μIU/mL
Creatinine Kinase (CK)	18 U/L (low)	38–190 U/L

Blood cultures were drawn and grew *Lacticaseibacillus casei*. Steroids/immunosuppressive treatment was deferred, and the infectious diseases (ID) team was consulted. The patient denied any raw foods, cheeses/yogurts, or consumption of probiotics. He denied ever having fevers or a history of rheumatic fever. The exam was notable for a new systolic murmur. He was initially started on intravenous vancomycin and ceftriaxone. Repeat blood cultures demonstrated persistent bacteremia, and he was switched to ampicillin‐sulbactam, 3 g every 24 h. Sensitivities demonstrated the species was sensitive to penicillin (minimum inhibitory concentration of 0.5 mcg/mL) and he was switched to renally adjusted ampicillin, 2 g every 12 h. Repeat blood cultures remained negative. Repeat TTE showed no changes or new vegetations. With his intermittent dialysis sessions and treatment of his *Lacticaseibacillus casei* endocarditis, the patient had gradual clinical improvement and was eventually weaned off dialysis. His renal function improved with a creatinine of 2.9 mg/dL. He otherwise remained stable and was discharged to rehabilitation, where he would complete a 6‐week antibiotic course with IV ampicillin. A follow‐up TTE 1 year later at an outside hospital did note a mitral vegetation; however, the patient was not a surgical candidate due to his age and medical comorbidities.

## 3. Discussion

*Lactobacillus casei* is a facultative anaerobic Gram‐positive rod, typically part of the normal gastrointestinal and oral microbiota; it is an unusual cause of IE, often associated with prosthetic valves, dental procedures, or immunosuppression. Their low virulence and indolent course may contribute to delayed recognition, particularly in patients who lack classic features of infection such as fever or leukocytosis, as seen in this patient. A key feature of this case was the diagnostic challenge posed by the patient’s presentation. The initial clinical picture was dominated by worsening renal function in the context of newly diagnosed valvular disease, without clear evidence of infection. This led to an initial attribution of renal injury to cardiorenal syndrome. Only after progression to dialysis‐dependent renal failure and biopsy‐proven focal necrotizing/crescentic GN was further workup done, ultimately revealing persistent bacteremia with *Lactobacillus casei*. This underscores the importance of maintaining a broad differential diagnosis in patients with unexplained GN, particularly when structural cardiac abnormalities are present as in the case of our patient.

IE‐associated RPGN, accounts for 3.4% (20/595) of cases with IE in a case series report, and it is mostly associated with *Staphylococcus aureus* and *Streptococcus* species [22]. To the best of our knowledge, no cases have been reported with *Lactobacillus* species which are generally considered low‐virulence organisms and uncommon causes of endocarditis. With this case we confirm that not only highly virulent pathogens can cause RPGN as part of IE complications.

About 25% of patients with IE‐associated RPGN were initially considered to have idiopathic vasculitis (ANCA‐associated vasculitis or IgA vasculitis), highlighting the diagnostic challenge [22]. Distinguishing infection‐associated GN from primary autoimmune disease is critical, as management strategies differ substantially. Immunosuppressive therapy, which is a mainstay in primary vasculitis, may worsen outcomes in the setting of active infection. In contrast, targeted antimicrobial therapy alone may lead to resolution of renal injury from infectious etiologies [19]. In rare cases, combination therapy of antimicrobials and immunosuppressive regimens is needed to rescue renal function [22]. In our patient, initiation of appropriate antibiotic therapy resulted in clearance of bacteremia and significant recovery of renal function, allowing discontinuation of dialysis without the use of immunosuppressive therapy.

## 4. Conclusion

Our case of RPGN secondary to *Lactobacillus casei* associated IE emphasizes a few key principles. First, IE should be considered in patients with unexplained GN and concurrent valvular dysfunction. Second, persistent growth across multiple blood cultures of low virulence species should prompt consideration of a true intravascular source. Additionally, the absence of echocardiographic evidence of vegetations does not exclude the diagnosis of IE, particularly in early or atypical cases. Third, antimicrobial therapy should be prioritized in suspected infection‐associated RPGN, with immunosuppression reserved for select cases where there is inadequate response or strong evidence of an alternative etiology. Early recognition and appropriate antimicrobial therapy, along with management of GN, are crucial to preventing irreversible kidney damage [23].

## Author Contributions

Jason Chavez and Joseph Borick were involved in direct patient care and manuscript drafting. Fernando Pedraza Taborda contributed to the initial inpatient nephrology workup and edits to the manuscript. Paola Lichtenberger supervised the project and provided final approval of the manuscript.

## Funding

This manuscript received no external funding.

## Disclosure

All authors reviewed and approved the final manuscript.

## Consent

Written and verbal consent was obtained from the patient for publication of this manuscript.

Institutional Review Board approval was not required for this case report in accordance with institutional policies.

## Conflicts of Interest

The authors declare no conflicts of interest.

## Data Availability

The data supporting the findings of this study are available from the corresponding author upon reasonable request.
